# Polarized hyperspectral microscopic imaging system for enhancing the visualization of collagen fibers and head and neck squamous cell carcinoma

**DOI:** 10.1117/1.JBO.29.1.016005

**Published:** 2024-01-18

**Authors:** Ximing Zhou, Ling Ma, Hasan K. Mubarak, Doreen Palsgrove, Baran D. Sumer, Amy Y. Chen, Baowei Fei

**Affiliations:** a The University of Texas at Dallas, Department of Bioengineering, Richardson, Texas, United States; b The University of Texas at Dallas, Center for Imaging and Surgical Innovation, Richardson, Texas, United States; c The University of Texas Southwestern Medical Center, Department of Pathology, Dallas, Texas, United States; d The University of Texas Southwestern Medical Center, Department of Otolaryngology, Dallas, Texas, United States; e Emory University, Department of Otolaryngology, Atlanta, Georgia, United States; f The University of Texas Southwestern Medical Center, Department of Radiology, Dallas, Texas, United States

**Keywords:** polarized hyperspectral imaging, Stokes vector, head and neck cancer, squamous cell carcinoma, collagen

## Abstract

**Significance:**

Polarized hyperspectral microscopes with the capability of full Stokes vector imaging have potential for many biological and medical applications.

**Aim:**

The aim of this study is to investigate polarized hyperspectral imaging (PHSI) for improving the visualization of collagen fibers, which is an important biomarker related to tumor development, and improving the differentiation of normal and tumor cells on pathologic slides.

**Approach:**

We customized a polarized hyperspectral microscopic imaging system comprising an upright microscope with a motorized stage, two linear polarizers, two liquid crystal variable retarders (LCVRs), and a compact SnapScan hyperspectral camera. The polarizers and LCVRs worked in tandem with the hyperspectral camera to acquire polarized hyperspectral images, which were further used to calculate four Stokes vectors: S0, S1, S2, and S3. Synthetic RGB images of the Stokes vectors were generated for the visualization of cellular components in PHSI images. Regions of interest of collagen, normal cells, and tumor cells in the synthetic RGB images were selected, and spectral signatures of the selected components were extracted for comparison. Specifically, we qualitatively and quantitatively investigated the enhanced visualization and spectral characteristics of dense fibers and sparse fibers in normal stroma tissue, fibers accumulated within tumors, and fibers accumulated around tumors.

**Results:**

By employing our customized polarized hyperspectral microscope, we extract the spectral signatures of Stokes vector parameters of collagen as well as of tumor and normal cells. The measurement of Stokes vector parameters increased the image contrast of collagen fibers and cells in the slides.

**Conclusions:**

With the spatial and spectral information from the Stokes vector data cubes (S0, S1, S2, and S3), our PHSI microscope system enhances the visualization of tumor cells and tumor microenvironment components, thus being beneficial for pathology and oncology.

## 1 Introduction

Head and neck squamous cell carcinoma (HNSCC) is a major head and neck malignancy originating from the mucosal epithelium in the mouth, nose, and throat.^1^ HNSCC screening procedures usually start with a visual inspection and are followed by a biopsy, but the heterogeneous morphology of tissue and the small sampling area of biopsy determine that pathological examine is still needed to judge based on the cell morphology and other microscopic anatomical changes.^2^^,^^3^ Normally, the pathological diagnosis of HNSCC can be implemented with hematoxylin and eosin (H&E)-stained slides with a regular microscope. Certain features that are related to HNSCC, including keratinization, atypical mitoses, and enlarged nuclei size, can be clearly observed in RGB histology images of the H&E-stained slides.^4^^,^^5^ However, the tumor microenvironment consists of multiple biochemical, mechanical, and structural signals, and some of the major structural components, such as collagen,^6^^,^^7^ are not obviously noticeable in those images. Collagen tends to accumulate in and around tumors during cancer development and growth.^8^ Understanding its role in both normal and abnormal functions is beneficial for oncology study.^7^ Multidimensional optical imaging has grown rapidly in recent years. Rather than measuring only the two-dimensional (2D) spatial distribution of light as in the conventional photography, multidimensional optical imaging captures unprecedented information about photons’ spatial coordinates, emittance angles, wavelength, time, and polarization.^9^ Therefore, it has shown great potential for the study of microenvironment biomarkers. Two-photon microscopy,^10^ Raman microspectroscopy,^11^ and fluorescence lifetime imaging^12^ are some examples that can be used to explore collagen and other cellular components; however, they all require expensive and complex optics, which makes it difficult to incorporate these technologies into the standard pathological analysis process.

Hyperspectral imaging (HSI) is an optical imaging method that was originally used in remote sensing and has been applied to many other fields including biomedical applications.^13^ It acquires a spectral signature at every pixel in a 2D image and constructs a three-dimensional (3D) data cube, in which rich spatial and spectral information can be obtained simultaneously. HSI has been implemented for the detection of head and neck cancer.^14^ Yushkov and Molchanov^15^ developed an acoustic-optic HSI system with an amplitude mask that improved the contrast for phase visualization in the stained and unstained histological sections of human thyroid cancer. Our group has investigated several machine learning and deep learning algorithms for head and neck cancer detection in histological slides based on HSI and proved the usefulness of HSI. We carried out HNSCC detection based on the morphology and spectral signatures of the nuclei, respectively, and we found that both spatial and spectral information have significant impacts on the classification results.^4^ Using an inception-based deep neural network, we implemented whole-slide HNSCC detection and proved that HSI outperforms RGB^16^^,^^17^ Furthermore, by employing a pre-trained video transformer on hyperspectral microscopic data, an detection accuracy of 89.64% was achieved in whole-slide thyroid cancer histology images.^18^^,^^19^ With the development of a fully automated HSI microscope, HSI can be easily adapted into routine pathology analysis.^20^^,^^21^

Polarized light imaging is an effective optical imaging technique for exploring the structure and morphology of biological tissues through obtaining their polarization characteristics.^22^ It can acquire the 2D spatial polarization information of the tissue, which reflects various physical properties of the tissue, including surface texture, surface roughness, and surface morphology information.^23^^–^^27^ The categories of polarized light imaging techniques, namely linear polarization imaging,^3^^,^^28^^,^^29^ Muller matrix imaging,^30^^,^^31^ and Stokes vector imaging,^32^ have been applied for head and neck cancer detection. An orthogonal polarization spectral imaging method, which is a type of linear polarization imaging method, was implemented for the evaluation of antivascular tumor treatment and oral squamous cell carcinoma on tissue.^28^^,^^29^ A multispectral digital microscope with an orthogonal polarized reflectance imaging mode was developed for *in vivo* detection of oral neoplasia.^3^ A 4×4 Muller matrix imaging and polar decomposition method were applied for diagnosis of oral precancer.^31^ Researchers also adopted a 3×3 Muller matrix imaging method for oral cancer detection.^30^

Polarized HSI (PHSI) is a combination of polarization measurement, spectral analysis, and space imaging technology. It obtains the polarization, spectral and morphological information of the object simultaneously^33^^–^^35^ and thus provides more image information for digital pathology compared with RGB histology images. In our previous study, we developed a dual-modality optical imaging microscope by combining HSI and polarized light imaging, and we reported our preliminary study on the use of the PHSI microscope for distinguishing squamous cell carcinoma from normal tissue on H&E stained slides based on the spectra of the Stokes vector.^32^^,^^36^ Setting up our polarized hyperspectral microscope only requires mounting the polarization components, which are assembled in a compact cage system, on the conventional optical microscope and replacing the RGB camera with a hyperspectral camera. Therefore, our customized microscope can more easily be incorporated into the standard pathological analysis process to work with the existing conventional optical microscopes.

In this paper, we apply our polarized hyperspectral microscopic imaging system to acquire images of HNSCC histological slides to visualize collagen fibers and squamous cells in both normal and cancerous tissues. To the best of our knowledge, this is the first work of applying PHSI to improve the visualization of collagen based on the spatial and spectral information in Stokes vector data cubes and using PHSI to differentiate normal cells and tumor cells on HNSCC pathologic slides based on Stokes vector parameters.

## 2 Methods

### 2.1 Polarized HSI

**Fig. 1:**
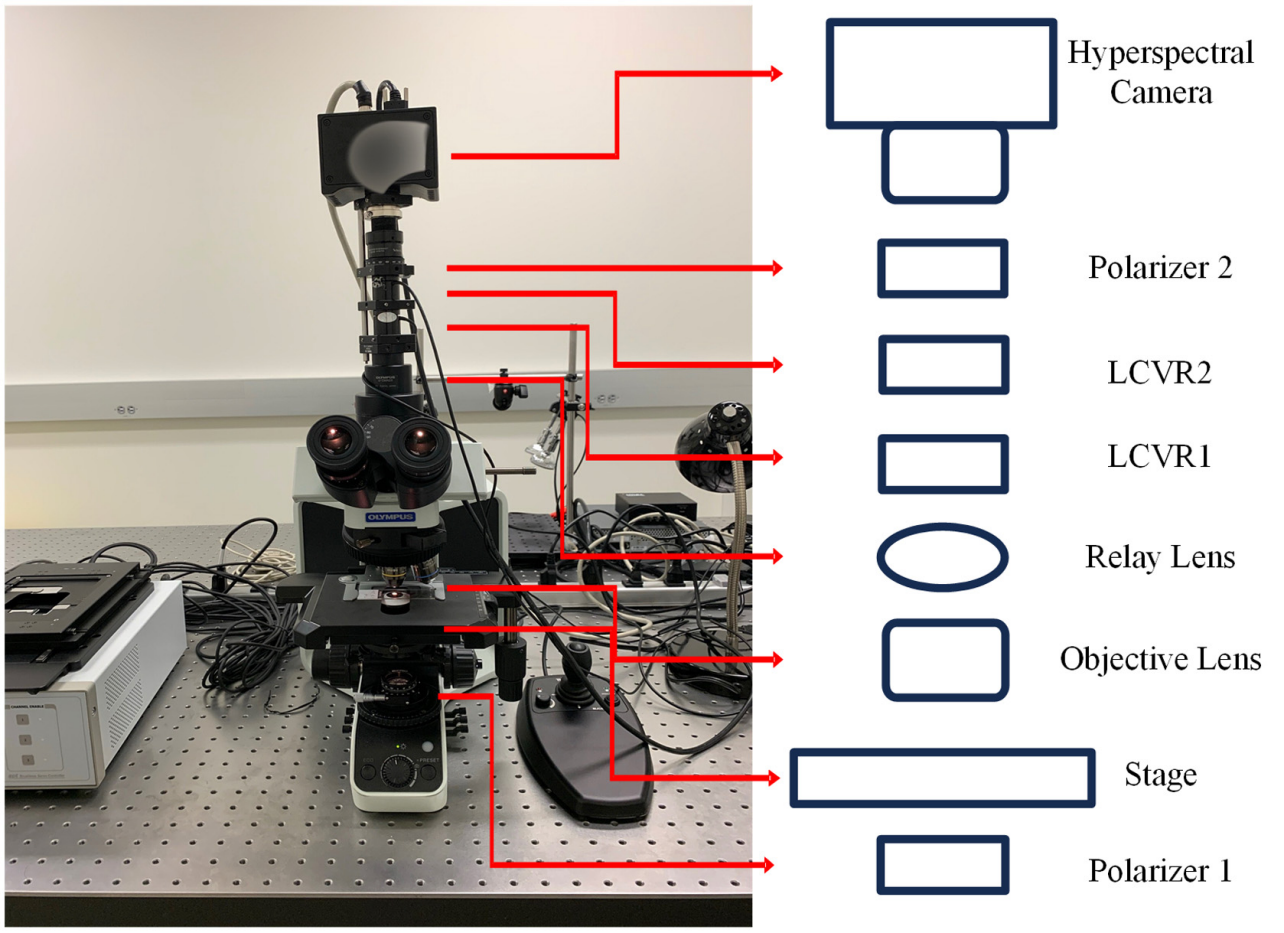
The setup of the PHSI microscope system. The transmissive axis orientation of polarizer 1 was set at 45 deg, and that of polarizer 2 was set at 0 deg. The fast axis orientation of LCVR1 was set at 0 deg, and that of LCVR2 at 45 deg.

In this study, we employ a customized polarized hyperspectral microscope, which is capable of full Stokes polarized hyperspectral imaging.^32^ The core components of the imaging system include an upright optical microscope with an integrated halogen light source, two linear polarizers, two liquid crystal variable retarders (LCVRs), and a customized SnapScan hyperspectral camera. The LCVRs and polarizers are for polarized light imaging. The transmissive axis of polarizer 1 was set at 45 deg, and the transmissive axis of polarizer 2 was at 0 deg. The fast axis of LCVR 1 was set at 0 deg, and that of LCVR 2 was at 45 deg. The SnapScan hyperspectral camera was able to acquire data through the translation of the imaging sensor inside of the camera. It had a wavelength range of 470 to 750 nm with 97 spectral bands and an image resolution of 1200×1200  pixels. The polarized light imaging components and HSI components worked together to acquire images in the visible-infrared wavelength range. Images were collected under 10× magnification and 40× magnification. Figure 1 demonstrates the setup of the imaging system with transmissive axis orientations of the polarizers and the fast axis orientations of LCVRs.

**Fig. 2:**
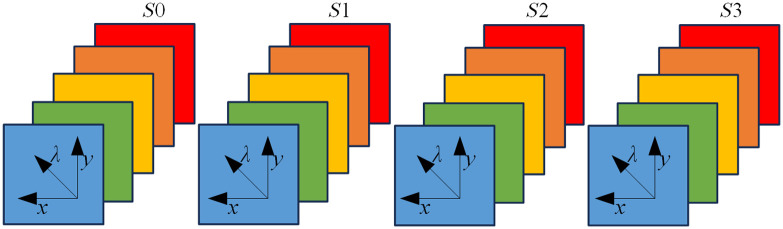
Diagram of full-polarization Stokes vector data cubes, each of which has three dimensions including two spatial dimensions (x, y) and one spectral dimension (λ).

The system is capable of full Stokes polarimetric imaging by acquiring four element images, namely $I_{h}$, $I_{v}$, $I_{45}$, and $I_{rc}$. $I_{h}$ represents the light intensity measured with a horizontal linear analyzer, in which the retardations of LCVR 1 and LCVR 2 are both set to 0 rad; $I_{v}$ represents the light intensity measured with a vertical linear analyzer, in which LCVR 1 is set at 0 rad retardation and LCVR 2 is set at π rad retardation; $I_{45}$ represents the light intensity measured with a 45 deg oriented linear analyzer, in which LCVR 1 and LCVR 2 are both set at π/2 rad retardation; and $I_{rc}$ represents the light intensity measured with a right circular analyzer, in which LCVR 1 is set at 0 rad retardation and LCVR 2 is set at π/2 rad retardation. The phase retardation of LCVR is determined by applying different values of voltage on it. In the PHSI dataset obtained by the system, each element image is a 3D data with two spatial dimensions and one spectral dimension. After image acquisition, the Stokes vectors (S0, S1, S2, and S3) were calculated using the four element images, as defined in Eq. (1). Each Stokes parameter was also a 3D data cube, as shown in Fig. 2. In addition, the value of S0 is equal to the value of total light intensity.

$$S0=I_{h}+I_{v},\;S1=I_{h}-I_{v},\;S2=2\times I_{45}-(I_{h}+I_{v}),\;S3=2\times I_{rc}-(I_{h}+I_{v}).$$ (1)

### 2.2 Sample Preparation

Fresh surgical tissue samples were obtained from patients who underwent surgical resection of head and neck cancer, as we described earlier.^37^ For each patient, a sample of the primary tumor, a normal tissue sample, and a sample at the tumor-normal margin were collected. Fresh *ex vivo* tissues were formalin fixed, paraffin embedded, sectioned, stained with H&E, and digitized using whole-slide scanner. Then, a board-certified pathologist specializing in head and neck cancer outlined the cancer margin in the digitized histology images. The annotations were used as the histologic ground truth in this study.

### 2.3 Synthetic RGB Images

**Fig. 3:**
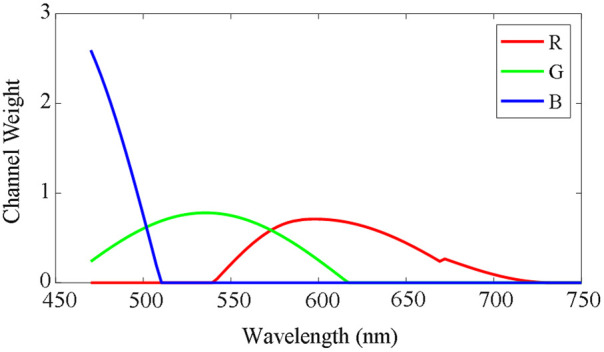
Transformation function to synthesize RGB images from Stokes vector data cubes. The weights for red (R), green (G), and blue (B) channels are modified specifically for our PHSI data.

To generate synthetic RGB images from Stokes vector data cubes, we adopted an HSI-to-RGB transformation function similar to the spectral response of human eyes and modified it specifically for our PHSI data,^4^^,^^38^ as shown in Fig. 3. In the transformation process, three spectral response curves (R, G, and B) multiply with the data cubes, respectively, to generate three channels (red, green, and blue) in the synthetic RGB images. We applied this HSI-to-RGB transformation to all four Stokes vector data cubes (S0, S1, S2, and S3) to generate four PHSI-synthesized RGB images. We used the synthetic RGB images of S0 as an H&E analog, which may not perfectly represent a standard visible light microscope.

### 2.4 System Calibration

We implemented the calibration strategy based on a quarter wave plate following a standard calibration method.^39^ We rotated the fast axis of the quarter wave plate from 0 deg to 180 deg with a 10-deg increment and acquired a set of four hyperspectral data cubes at each step. The theoretical relationships between the Stokes vector parameters (S1, S2, and S3) normalized by S0 and fast axis positions (θ) are shown in Eq. (2):

S1=sin(2θ)cos(2θ),S2=sin(2θ)sin(2θ),S3=−cos(2θ). (2)

We selected three wavelengths for calibration (470, 525, and 626 nm) and calculated the root mean square error (RMSE) for normalized S1, S2, and S3. The RMSE is defined as follows:

$$\mathrm{RMSE}=\mathrm{sqrt}(\sum_{n=1}^{N}((S(\exp\;\theta (n))-S{\left(\text{theory }\theta (n)\right)}^{2}),$$ (3)

where n indicates the index of rotation, which in this case is from 1 to 19 to match the rotation from 0 deg to 180 deg at 10-deg increment. θ(n) indicates the exact angle corresponding to the rotation index, “exp” refers to the experimental values of Stokes vector parameters, and “theory” refers to the theoretical values of the Stokes vector parameters.

In this study, 470 nm is the starting wavelength of the hyperspectral camera. 525 nm has a 55 nm increment from 470 nm, and 626 nm has a 156 nm increment from 470 nm. The three chosen wavelengths cover a large wavelength range of the data cubes; thus we believe that they can be representative to show that our system can accurately generate Stokes vector parameters within the wavelength range.

### 2.5 Contrast of Collagen

The metric that we introduce in this study to evaluate the imaging of collagen is contrast, which is determined by the difference in the color and brightness of the object and other objects within the same field of view (FOV). The calculation of contrast is expressed as

Contrast=∑r(i,j)*r(i,j)*p(i,j), (4)

where r(i,j) represents the gray level difference between the adjacent pixels at location i and j and p(i,j) represents the probability distribution of r(i,j).

### 2.6 Spectra Extraction

To accurately extract the spectra of Stokes vector related parameters from collagen fibers, normal squamous cells, and tumor cells, we manually outlined the regions of interest (ROIs) in the synthetic RGB images of S0 to generate the binary masks of collagen fibers, normal cells, and tumor cells. Because the FOVs of four Stokes vector cubes were identical, we applied the same binary masks to all four data cubes of Stokes vector parameters to extract the pixel-level spectra of the ROI and calculated the mean and standard deviation of all pixels in the ROI. This process helps to remove the influence of background pixels on the spectra of Stokes vector parameters.

## 3 Results

### 3.1 Calibration of the Polarized Hyperspectral Microscope

**Fig. 4:**
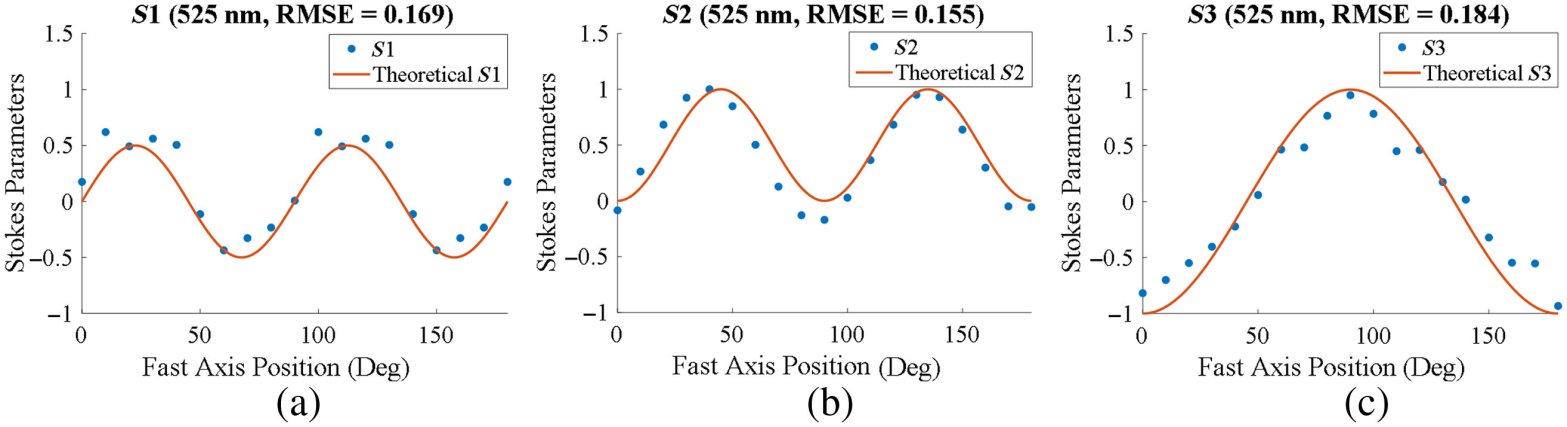
Calibration results of three Stokes vectors under 525 nm. The theoretical curves of S1, S2, and S3 and the fast axis positions are plotted along with the scattered plots of the experimental values of S1, S2, and S3 and fast axis positions: (a) calibration result of Stokes vector S1 with an RMSE of 0.169, (b) calibration of Stokes vector S2 with an RMSE of 0.155, and (c) calibration of Stokes vector S3 with an RMSE of 0.184.

We carried out system calibration and calculated RMSE at three different wavelengths (470, 525, and 626 nm), as given in Table 1. All RMSE values were <0.2, which shows the satisfactory performance of our system. Figure 4 shows the theoretical curve and experimental data of the relationship between Stokes parameters (S1, S2, and S3) and fast axis positions at 525 nm. It can be seen that the experimental data agree with the theoretical curves of S1, S2, and S3.

**Table 1:** RMSE of Stokes vectors S1, S2, and S3 at 470, 525, and 626 nm, respectively.

Wavelength (nm)	S1	S2	S3
470	0.155	0.149	0.191
525	0.169	0.155	0.184
626	0.196	0.178	0.172

### 3.2 Enhanced Visualization of Collagen in Polarized Hyperspectral Images

**Fig. 5:**
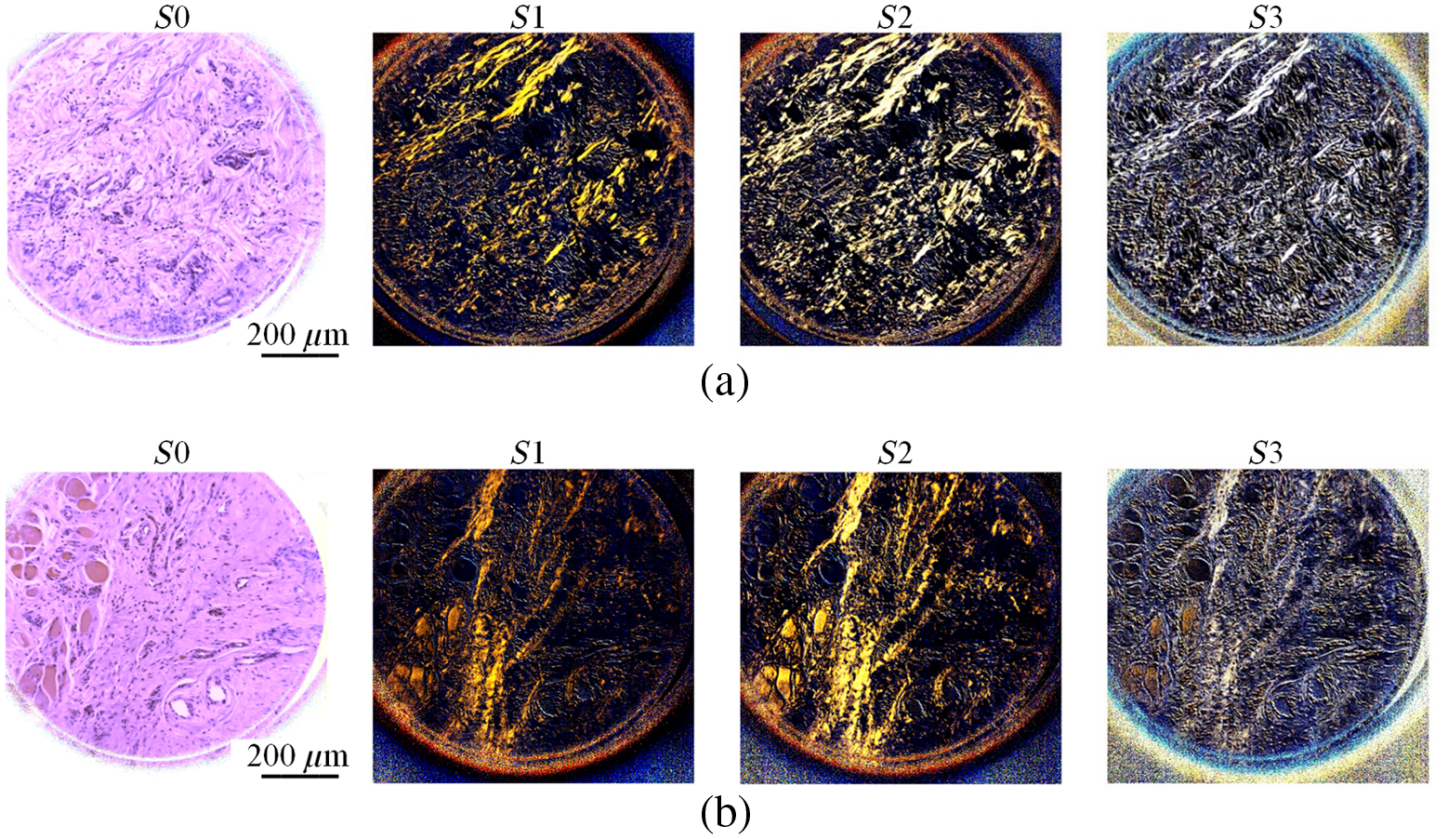
Synthesized RGB images generated using four Stokes vectors (S0, S1, S2, and S3) from two different regions in normal stroma tissue: (a) normal stroma containing both dense and sparse fibers and (b) normal stroma with sparse fibers.

**Fig. 6:**
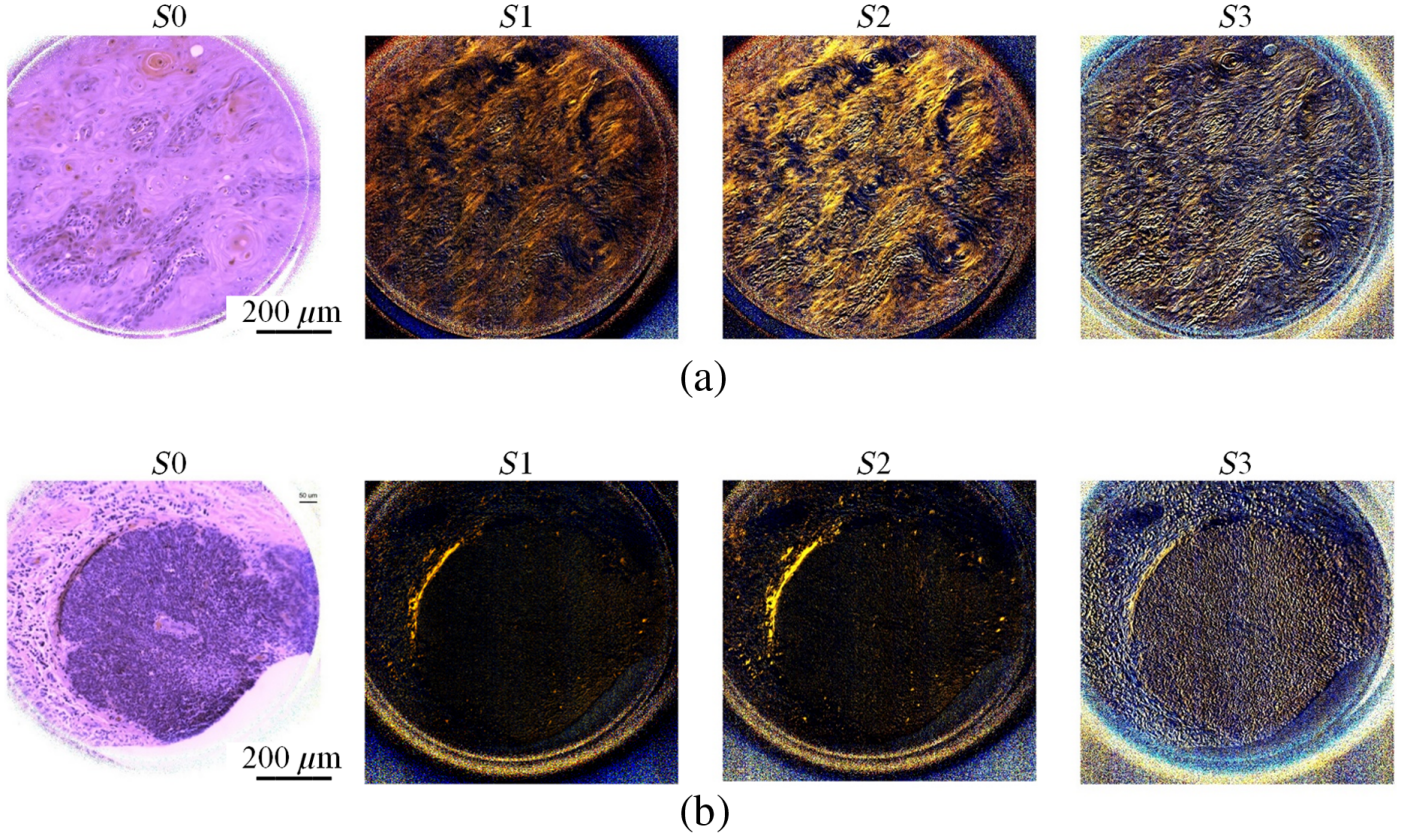
Synthesized RGB images of S0, S1, S2, and S3 from two cancerous regions containing fibers: (a) cancerous tissue with fibers accumulated in tumor and (b) cancerous tissue with fibers accumulated around tumor.

In this section, we qualitatively and quantitatively demonstrate the enhanced visualization of collagen fibers in histological slides using PHSI. Figure 5 shows the PHSI-synthesized RGB images of two regions in normal tissues. Each synthetic RGB was generated using one Stokes vector parameter, and each set of four Stokes parameter images share the same FOV. The normal stroma tissue in Fig. 5(a) contains both dense and sparse collagen fibers. We found that dense collagen fibers, which are mostly located at the bottom region of the images, could be observed in all four Stokes vector parameters. In addition, the sparse collagen fibers in the upper side of the images were difficult to be recognized in S0, but they were easily recognized in S1, S2, and S3. Figure 5(b) shows another region in normal stroma tissue containing sparse collagen fibers. Similarly, it is hard to identify the regions containing sparse collagen fibers in S0, but the distribution of sparse collagen fibers can be seen clearly in S1, S2, and S3. Figure 6 shows the synthesized RGB images of the Stokes vector parameters of two cancerous tissue regions containing collagen fibers. Specifically, Fig. 6(a) shows a region where fibers accumulated within the tumor, and in Fig. 6(b) the fibers were around the tumor. Similar to the images in Fig. 5, it is difficult to identify the sparse collagen fibers in S0, but their distribution can be observed clearly in S1, S2, and S3.

Contrast was calculated on all images with collagen shown in Figs. 5 and 6, and the values are given in Table 2. All images were saved in the double format, so the values of contrast are between 0 and 1. It can be seen that there is a significant increase of contrast on all images from S0 to S1, S2, and S3 in a sequence.

**Table 2:** Contrast for S0, S1, S2, and S3 of images with collagen under different conditions.

Collagen condition	S0	S1	S2	S3
Normal dense fibers	0.003	0.054	0.082	0.136
Normal sparse fibers	0.003	0.052	0.078	0.125
Fibers within tumor cells	0.006	0.056	0.088	0.126
Fibers around tumor cells	0.003	0.047	0.075	0.133

### 3.3 Spectral Analysis of Collagen Using PHSI

**Fig. 7:**
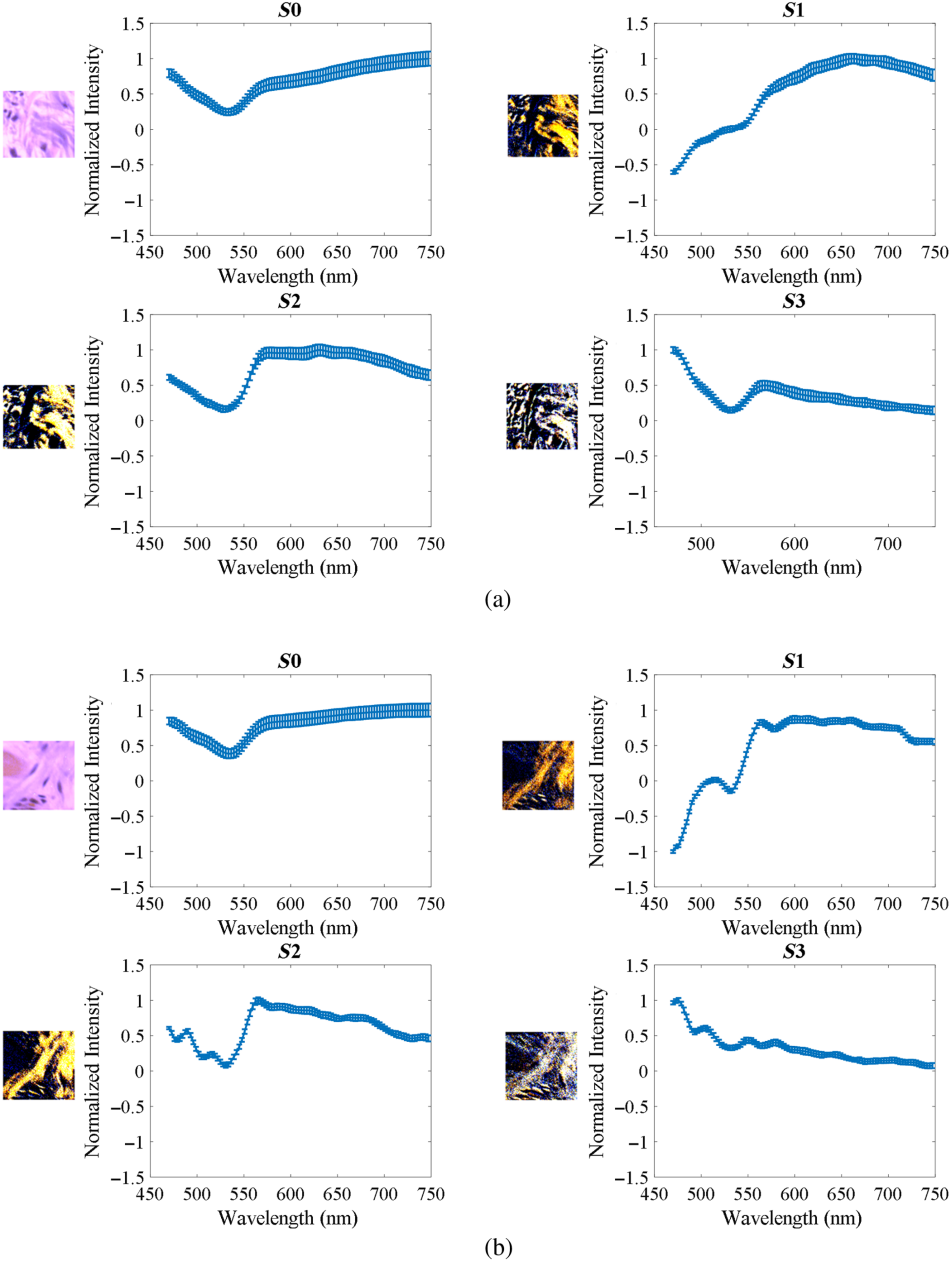
Synthesized RGB and average spectra of Stokes vectors from a small region in normal tissue: (a) normal tissue containing dense fibers and (b) normal tissue containing sparse fibers.

To further investigate the usefulness of PHSI for collagen study, we extracted spectra of Stokes vector parameters of collagen and calculated their mean and standard deviation. Figure 7(a) shows the PHSI-synthesized RGB images of a small region in normal tissue containing dense collagen fibers as well as the corresponding spectra of four Stokes parameters S0, S1, S2, and S3. We find a low peak near 550 nm appearing in the spectra of all four Stokes parameters. In addition, there is a common high peak on the spectra of S1, S2, and S3 near 575 nm. Figure 7(b) shows the synthesized RGB images and spectra of Stokes vector parameters of a small normal tissue region containing sparse fibers. Similarly, we can see the low peaks at 550 nm in the spectra of S0, S1, S2, and S3 and the high peaks at 575 nm on the spectra of S1 and S2. However, as there are more fluctuations on the spectra of S3, the high peak at 575 nm is not very clear to be seen.

**Fig. 8:**
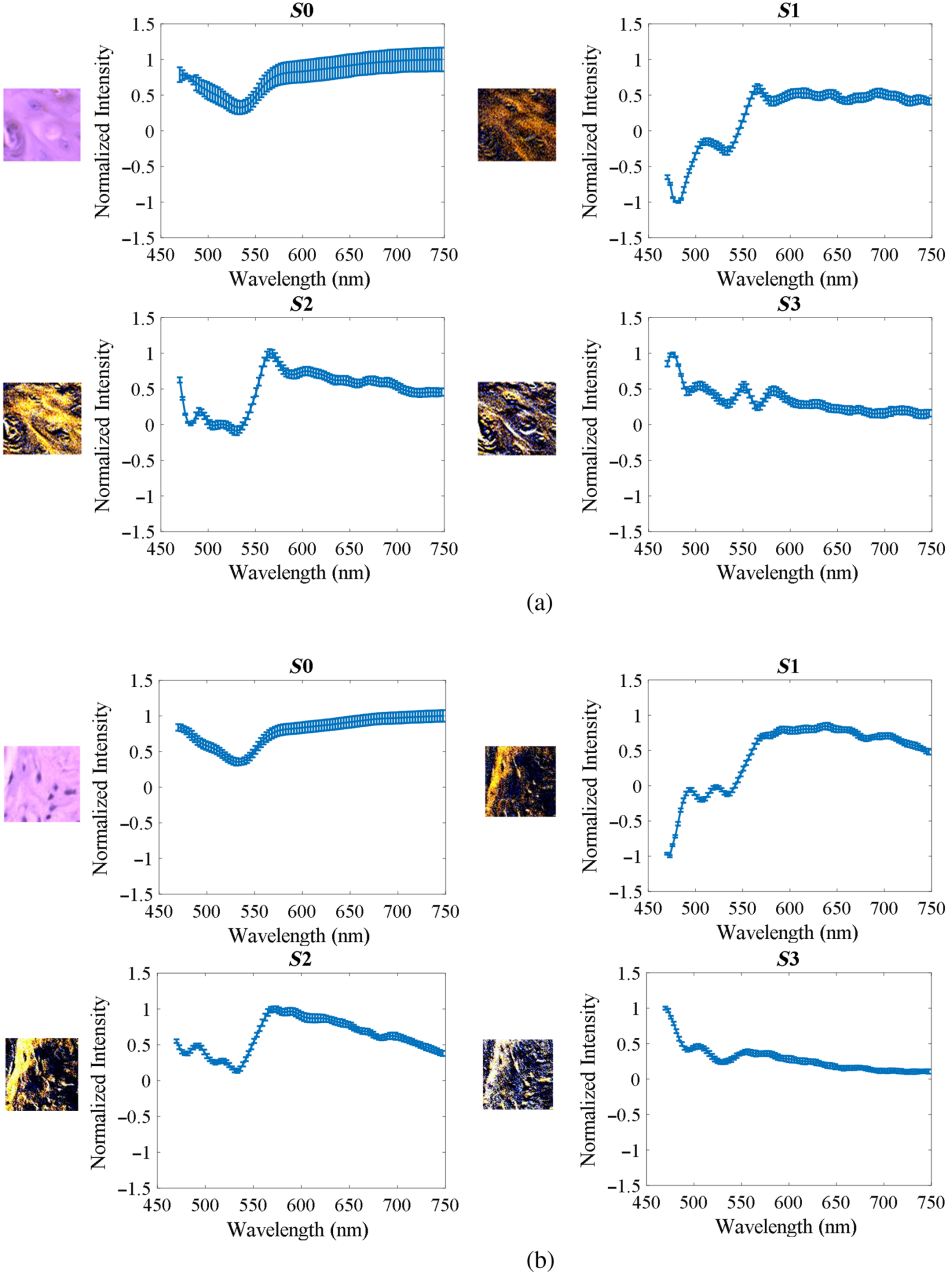
Synthesized RGB and average spectra of Stokes vectors from a small region in cancerous tissue: (a) tumor tissue with fibers growing within and (b) fibers around the tumor.

**Fig. 9:**
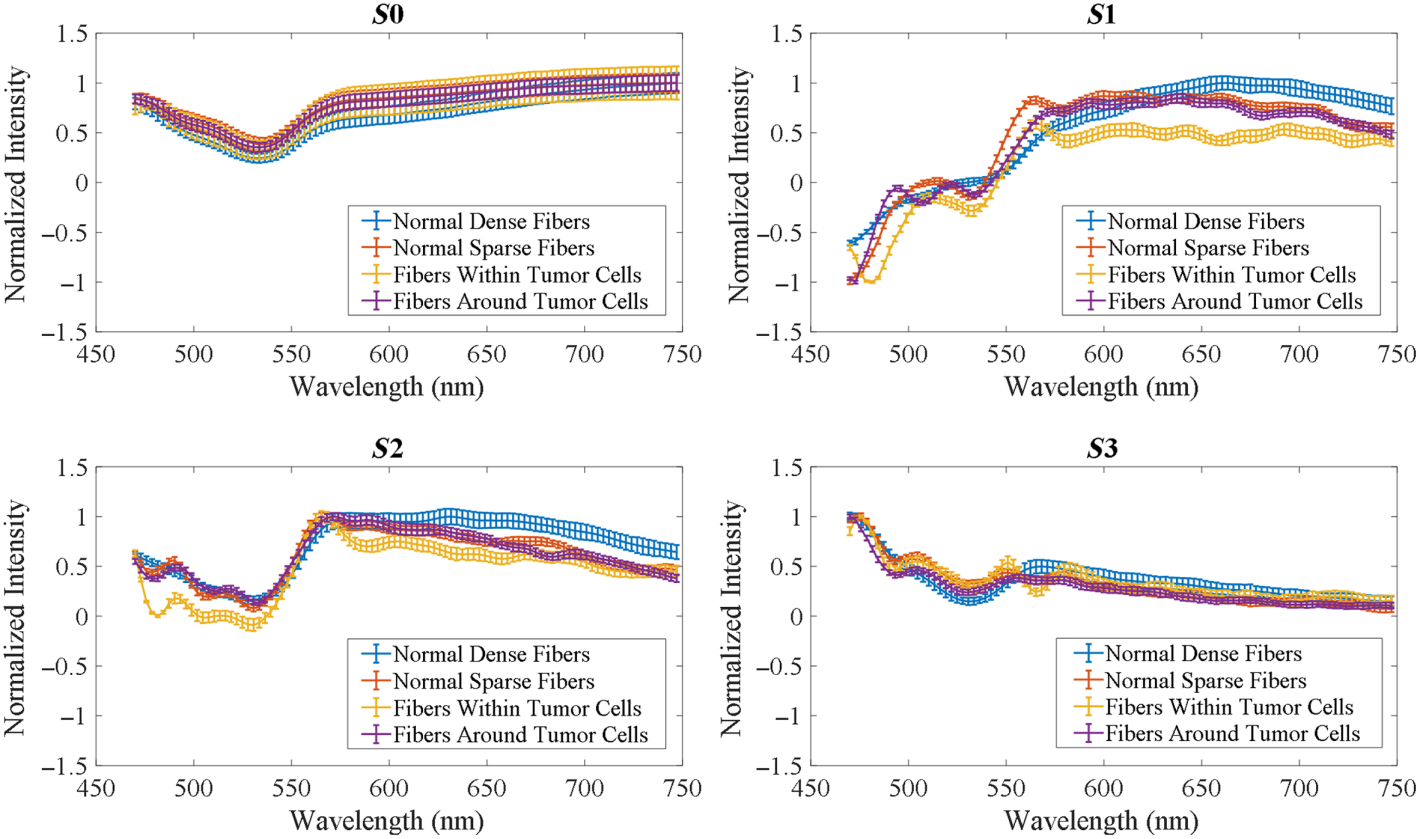
Spectral comparison of different fibers in Figs. 7 and 8.

Figure 8(a) shows the PHSI-synthesized RGB images and spectra of Stokes vector parameters of a small tumor tissue region containing fibers growing within the tumor. The spectra shape of S0, S1, and S2 are consistent with what we see in Fig. 7. For the spectra of S3, the low peak at 550 nm can be observed with more fluctuations. Figure 8(b) shows a small tumor region containing fibers growing around tumor cells. The spectra shapes of S0, S1, and S2 are consistent with what we find in the previous figures. For S3, we can observe a more significant increase after 550 nm. Figure 9 demonstrates the combination of the spectra (S0, S1, S2, and S3) in Figs. 7 and 8.

### 3.4 PHSI-Enhanced Visualization of Normal and Tumor Cells

**Fig. 10:**
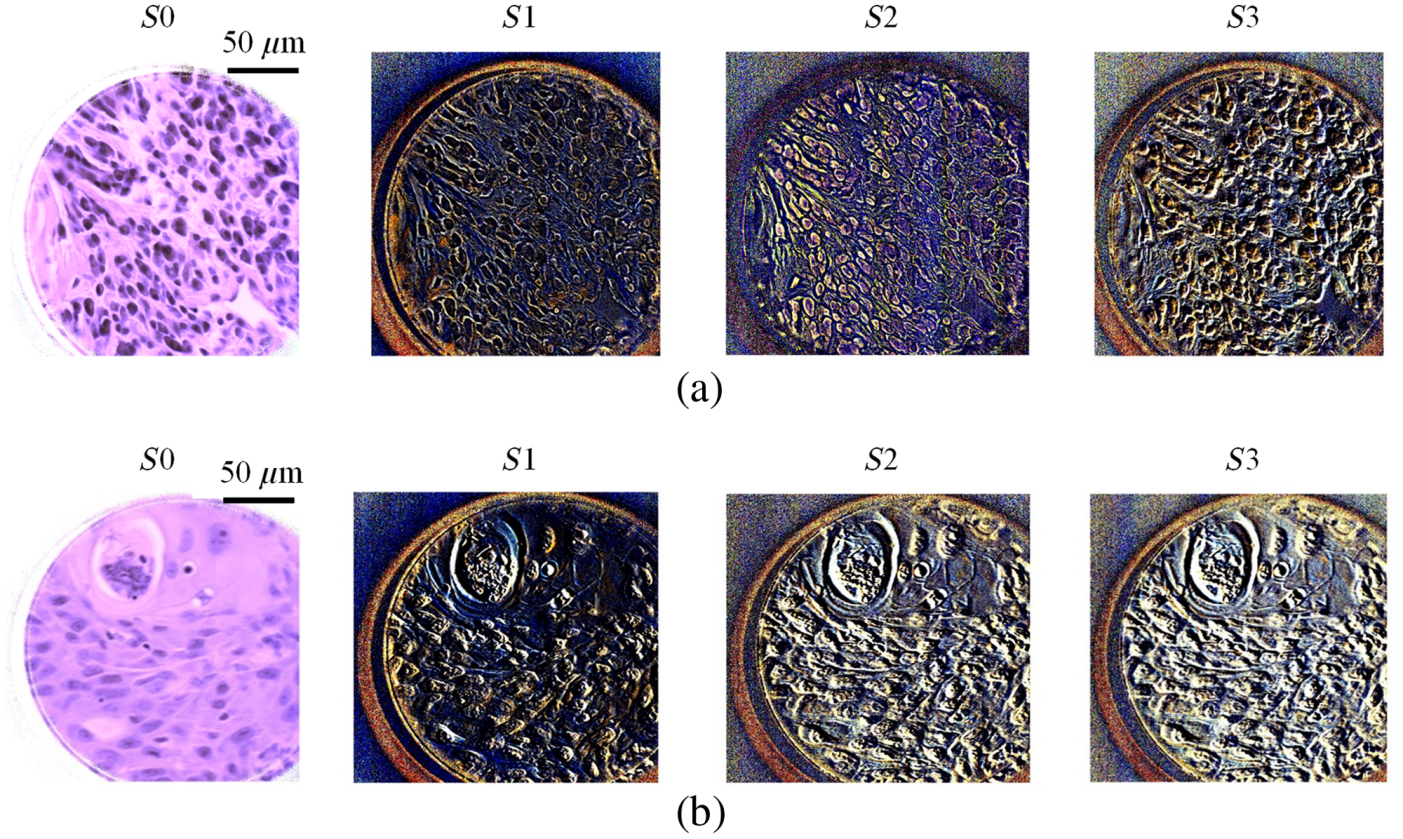
Synthesized RGB images generated from Stokes vectors of normal and tumor tissues: (a) normal tissue from a normal slide and (b) cancerous tissue from a tumor slide.

Figure 10(a) demonstrates the synthetic RGB images of Stokes vector parameters (S0, S1, S2, and S3) from a normal tissue region. We find that S1 and S2 enhance the image signal from the connective tissue, and S3 enhances the image signal from the normal cells. The 50  μm scale bar is specified on S0. Figure 10(b) demonstrates the synthetic RGB images of Stokes vector parameters (S0, S1, S2, and S3) from a tumor tissue region. This piece of tumor tissue contains a number of not well-differentiated tumor cells, featured by irregular or elliptical nuclei with coarse heterochromatin aggregates, increased fragmentation, or budding of nuclei.^40^^,^^41^ We find that S1, S2, and S3 help to improve the visualization of tumor cell morphology.

### 3.5 PHSI Spectra of Normal and Tumor Cells of HNSCC

**Fig. 11:**
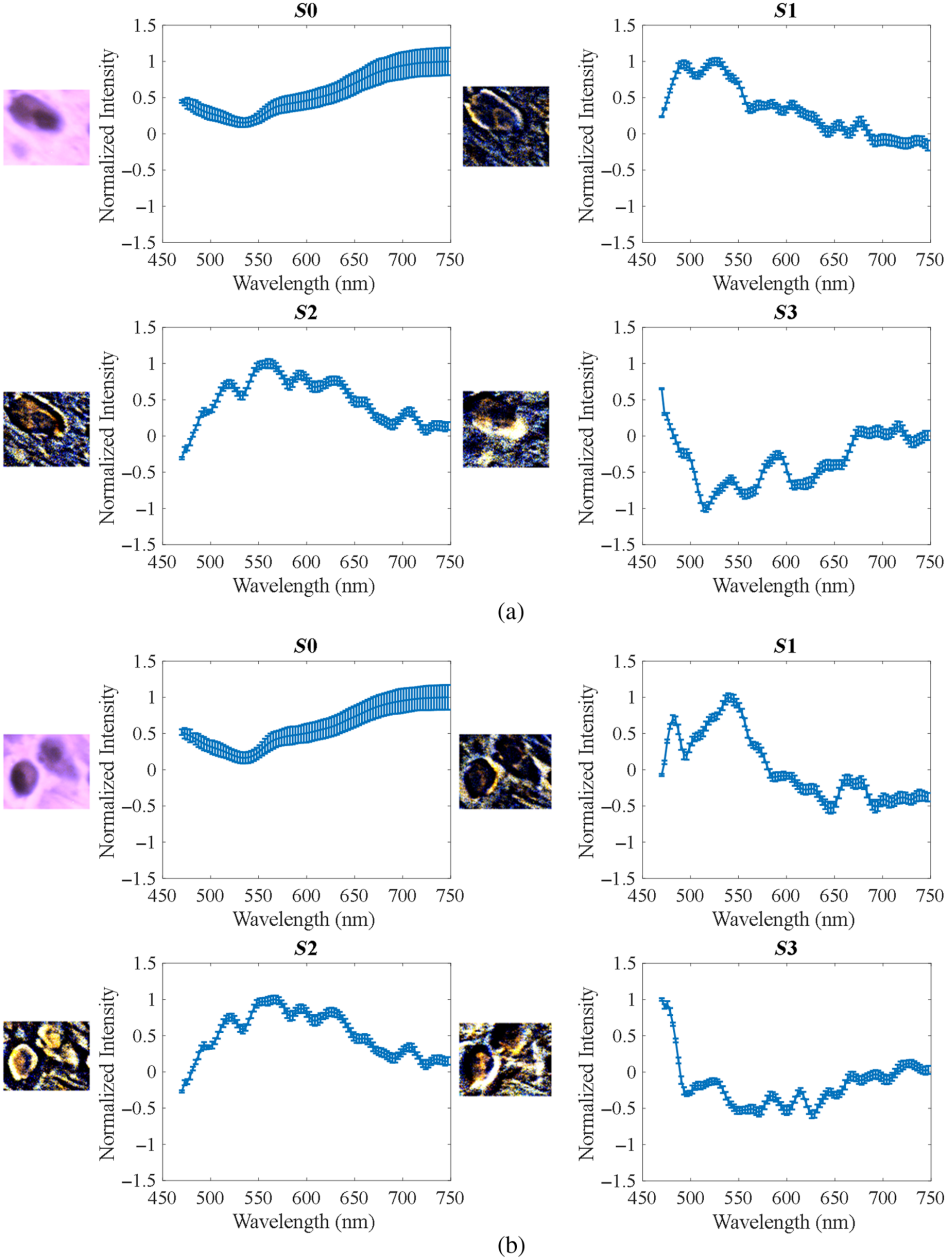
Synthesized RGB and Stokes vector spectra of single normal cell. In both (a) and (b), the spectra of four Stokes vectors show differences, and the nucleoli position is clearly observed.

**Fig. 12:**
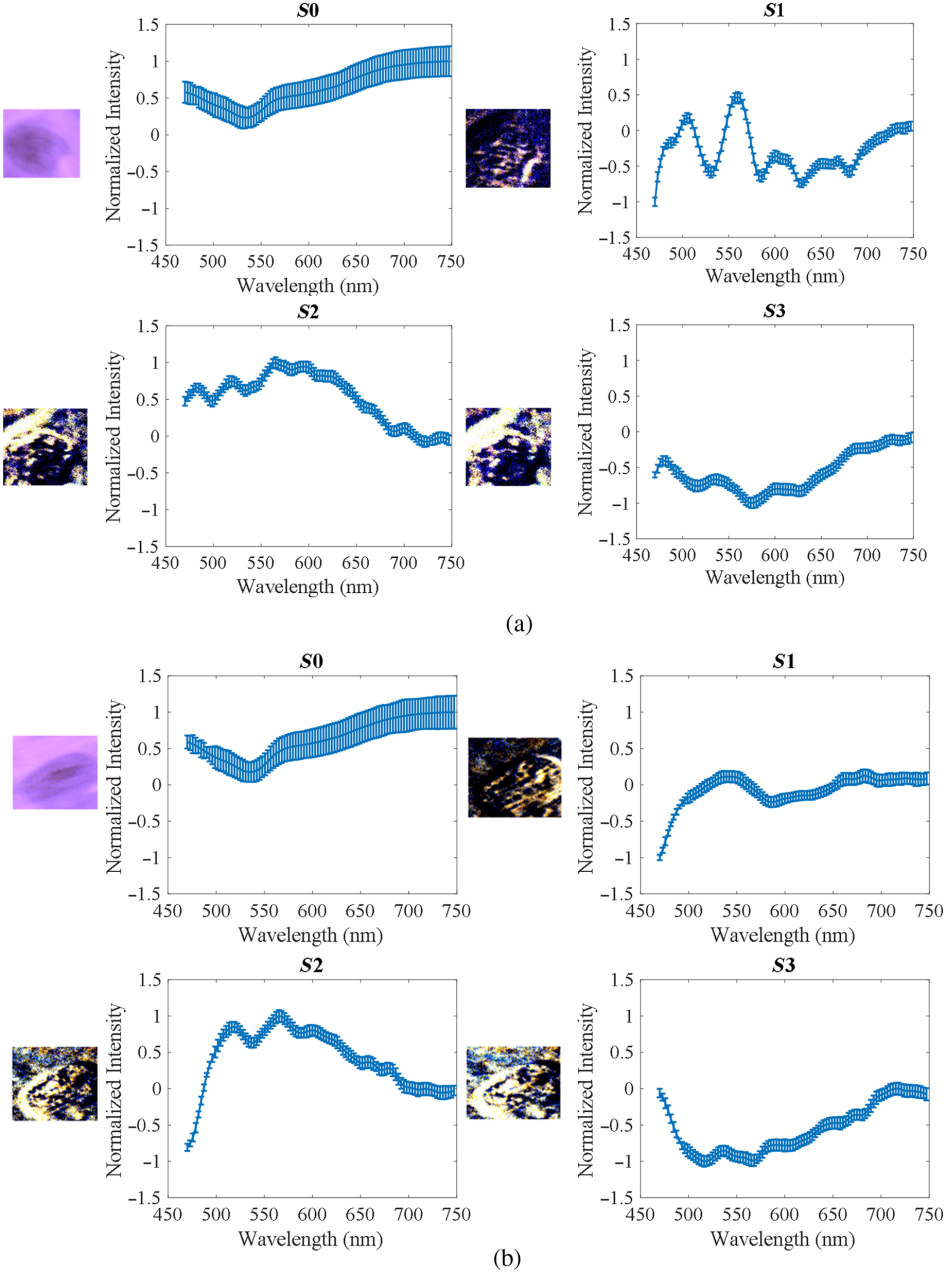
Synthetic RGB and Stokes vector spectra of tumor cells. In both (a) and (b), the spectra of four Stokes vectors show differences, and the nuclei morphology is clearly observed.

**Fig. 13:**
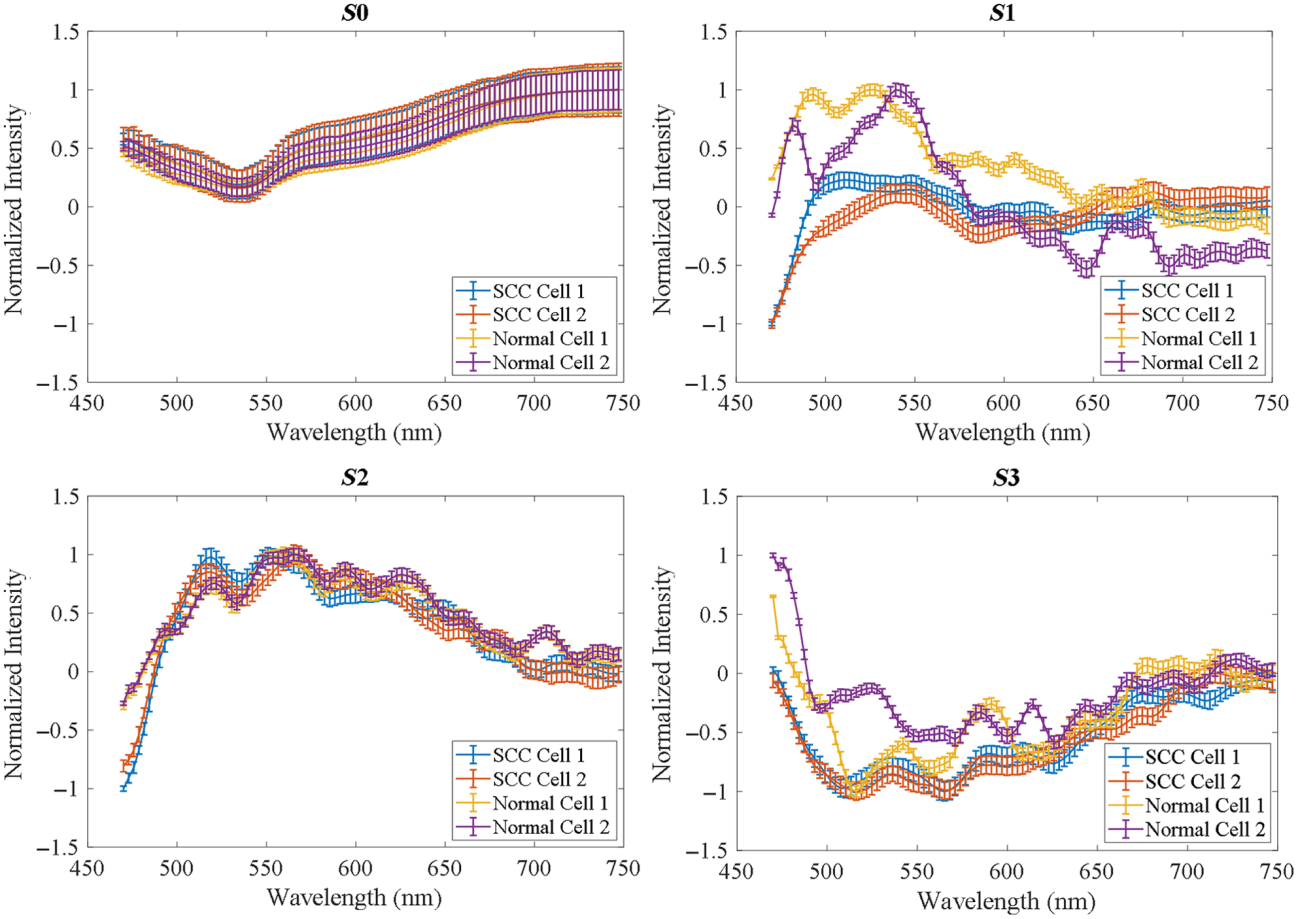
Spectra comparison between the selected two normal cells and two tumor cells on the HNSCC slides.

From the images acquired with our customized polarized hyperspectral microscope, we extracted the spectra of Stokes vector parameters of normal and tumor cells and calculated the mean and standard deviation. Figure 11 shows the PHSI-synthesized RGB images of Stokes vector parameters of two typical normal cells with a good differentiation of nucleus and cytoplasm structures, as well as the corresponding spectral curves of S0, S1, S2, and S3. The morphology of normal cell could be visualized clearly in all synthesized RGB images but is enhanced in S1 and S2. For the spectra of S0, a low peak near 550 nm is observed on both Figs. 11(a) and 11(b). For the spectra of S1, two close high peaks are observed near 475 nm and near 550 nm. For the spectra of S2, we observe a number of fluctuations, and the maximum normalized intensity appears near 550 nm. For the spectra of S3, there are also a number of fluctuations, with the minimum of normalized intensity appearing near 550 nm. Figure 12 demonstrates the PHSI-synthesized RGB images of Stokes vector parameters of two tumor cells and the corresponding spectra of S0, S1, S2, and S3. From Fig. 12(a), we find that it is hard to see the multiple nucleoli in S0, but we observe a good image contrast and morphological details of the nucleoli in S1, S2, and S3. The tumor cell in Fig. 12(b) is under a condition of increasing fragmentation of nuclei. It can be seen that the PHSI-synthesized RGB images of S1, S2, and S3 improve the visualization of nuclei fragmentation. The shape of the Stokes parameter spectra of two cells in Fig. 12 are consistent with each other. For S0, a low peak near 550 nm is observed. For S1, the two close high peaks near 475 nm and near 550 nm disappear. For S2, the two close high peaks near 475 and 550 nm tend to appear. For S3, there are several fluctuations. Figure 13 demonstrates the spectra comparison among two normal cells and two SCC cells. We find that the overlapping in the spectra of S0 and S2 is large among these cells. However, the spectra of S1 and S3 of the two SCC tumor cells do not overlap with those of the two normal cells, thus making the spectra of normal cells and tumor cells easier to be differentiated.

**Fig. 14:**
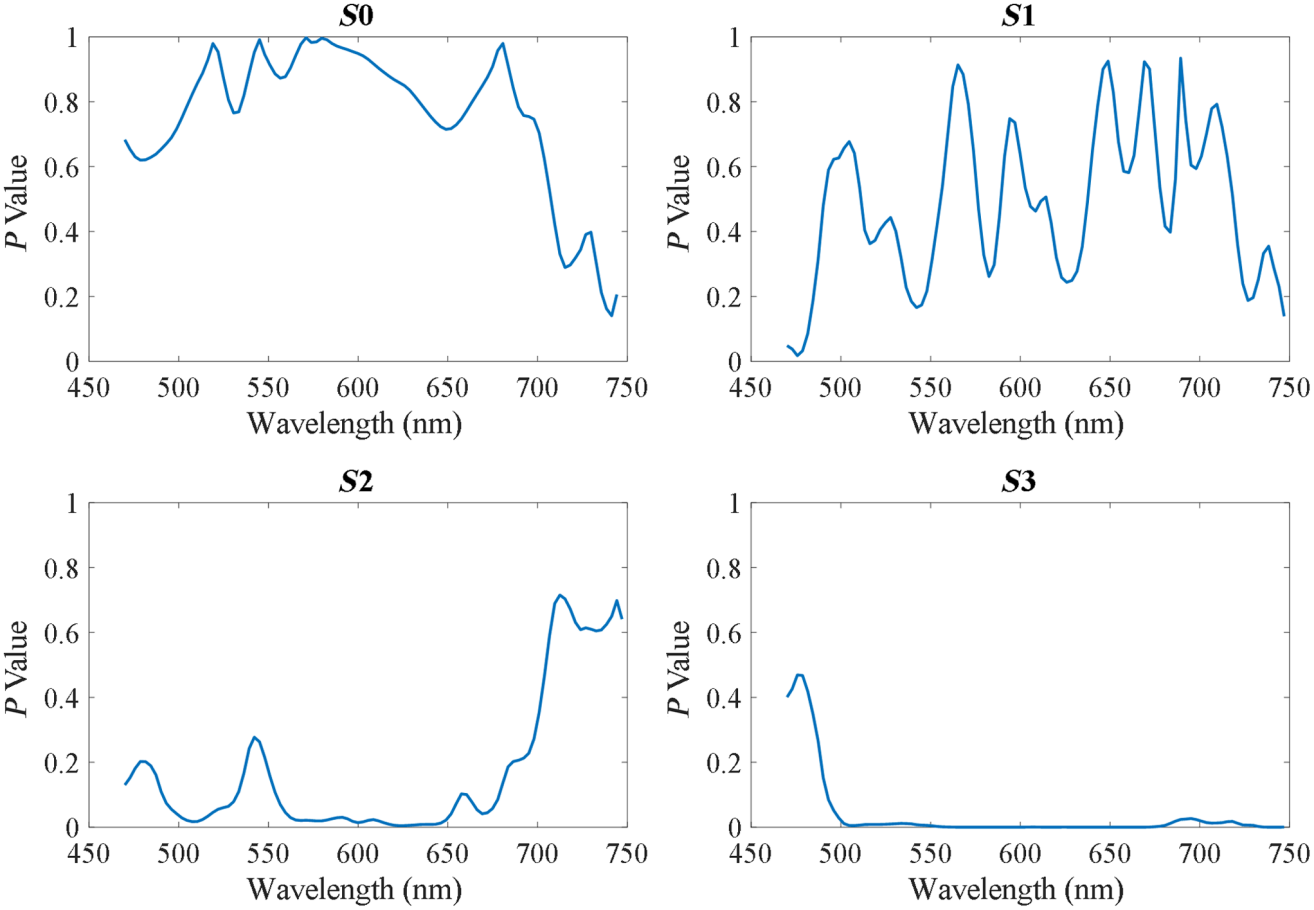
P values based on the two-sample t test among the average spectra of 15 normal cells and 15 tumor cells.

Furthermore, we implemented two-sample t test on the average spectra of Stokes vector parameters of 15 normal cells and 15 tumor cells, which were selected from three different patients (five normal cells and five tumor cells from each patient). The p values of different bands in each Stokes parameter are given in Fig. 14. It can be seen that the p values of S2 and S3 significantly decrease compared with those of S0, and S3 has p values <0.05 at most wavelengths. Note that the selection of these 30 cells did not strictly follow the assumption in standard statistical tests of statistically independent samples.

## 4 Discussion and Conclusion

In this study, we applied our customized polarized hyperspectral microscopic imaging system for enhanced visualization in both normal stroma tissues and cancerous tissues containing squamous cells and collagen fibers on HNSCC pathologic slides. Our results demonstrated that our PHSI microscope improves the visualization of collagen on HNSCC pathologic slides under different situations: dense fibers in normal stroma, sparse fibers in normal stroma, fibers accumulated in tumors, and fibers accumulated around tumors. Specifically, we find that the PHSI-synthesized images of S1, S2, and S3 are able to sensitively detect the fibrillar collagen on pathologic slides, especially on the regions with fibrillar collagen that cannot be clearly seen in S0 (similar to the images from RGB cameras), such as the sparse fibers in normal stroma and the fibers accumulated within tumors (cannot be seen clearly in S0). Our results suggest that S3 improves the contrast of collagen fibers compared with standard pathological examination, while also maintaining signal from small tumor cells. Our results also demonstrated that our PHSI microscope is capable of revealing the spectral signatures of collagen based on Stokes vector parameters (S0, S1, S2, and S3). In addition, our customized polarized hyperspectral microscope demonstrated its potential to better differentiate normal and tumor cells on HNSCC pathologic slides by increasing the contrast between the spectra of normal cells and tumor cells.

To the best of our knowledge, this is the first work to apply PHSI to improve the visualization of collagen based on the spatial and spectral information in Stokes vector data cubes. Although the results that we obtained so far in exploring the PHSI of collagen fibers are still preliminary, our customized polarized hyperspectral microscope has been proven to improve the visualization of collagen fibers and reveal the spectral signature of Stokes vector parameters of collagen fibers. In addition, this is also the first work to apply PHSI to differentiate normal cells and tumor cells on HNSCC pathologic slides. However, more work needs to be done for the quantitative analysis to study the Stokes vector parameter spectra of collagen growing under different situations and the spectra of Stokes vector parameters of normal cells and tumor cells. The PHSI technique can be further explored to study cancer diagnosis and prognosis based on collagen development in the future. Furthermore, incorporating PHSI with machine learning in the field of computational pathology is promising.

## Data Availability

Code and data underlying the results presented in this paper are not publicly available at this time but may be obtained from the authors upon reasonable request.
